# Aiming at Tobacco Harm Reduction: A survey comparing smokers differing in readiness to quit

**DOI:** 10.1186/1477-7517-3-13

**Published:** 2006-03-29

**Authors:** Maria Loumakou, Vasiliki Brouskeli, Jasmin-Olga Sarafidou

**Affiliations:** 1 University of Thessaly, Department of Primary Education, Argonafton – Filellinon st., 38221 Volos, Greece; 2 Labor Institute of the General Confederation of Greek workers, Sot. Petroula 5, 59100, Veria, Greece; 3 Institute of Child Health, Statistical Department, "Agia Sofia" Children's Hospital, Goudi, 11527, Athens, Greece

## Abstract

**Background:**

Greece has the highest smoking rates (in the 15-nation bloc) in Europe. The purpose of this study was to investigate Greek smokers' intention and appraisal of capability to quit employing the theoretical frameworks of Decisional Balance (DB) and Cognitive Dissonance (CD).

**Methods:**

A cross-sectional study including 401 Greek habitual smokers (205 men and 195 women), falling into four groups according to their intention and self-appraised capability to quit smoking was carried out. Participants completed a questionnaire recording their attitude towards smoking, intention and self appraised capability to quit smoking, socio-demographic information, as well as a DB and a CD scale.

**Results:**

The most numerous group of smokers (38%) consisted of those who neither intended nor felt capable to quit and these smokers perceived more benefits of smoking than negatives. DB changed gradually according to smokers' "readiness" to quit: the more ready they felt to quit the less the pros of smoking outnumbered the cons. Regarding relief of CD, smokers who intended but did not feel capable to quit employed more "excuses" compared to those who felt capable. Additionally smokers with a past history of unsuccessful quit attempts employed fewer "excuses" even though they were more frequently found among those who intended but did not feel capable to quit.

**Conclusion:**

Findings provide support for the DB theory. On the other hand, "excuses" do not appear to be extensively employed to reduce the conflict between smoking and concern for health. There is much heterogeneity regarding smokers' intention and appraised capability to quit, reflecting theoretical and methodological problems with the distinction among stages of change. Harm reduction programs and interventions designed to increase the implementation of smoking cessation should take into account the detrimental effect of past unsuccessful quit attempts.

## Background

Tobacco, the second major cause of death in the world, is currently responsible for the death of one in ten adults worldwide (about 5 million deaths per year) [1]. World Health Organization (WHO) estimates that, if current smoking patterns continue, it will cause some 10 million deaths every year by 2020, while half the people that smoke today will eventually be killed by tobacco [1]. Smoking is a prime factor in heart disease, stroke and chronic lung disease; it can cause cancer of the lungs, larynx, esophagus, mouth and bladder while it contributes to cancer of the cervix, pancreas, and kidneys [2]. Although numerous current studies document the health problems that smoking can cause, 23.5% of adults in the USA smoke [3]. Research evidence for smoking prevalence among adults in Greece at the same time is even more disappointing. As it has been shown, 37.6% of the adult population in Greece smokes [4]. The ATTICA study, that was conducted in year 2002 on the epidemiology of cardiovascular risk factors in Greece [5], revealed that 55% of males and 46.5% of females aged between 35–44 were daily smokers ( > 5 cigarettes/day). Furthermore, Eurostat figures regarding 1999 list Greece as having the highest smoking rates in the 15-nation bloc of Europe [6].

A great number of recent studies dealt with quitting smoking: preparation, efforts, stages, success and failure in trying to quit smoking are some of the favorite topics of the researchers today. The Transtheoretical Model (TTM) [7,8] is a widely used model of behavior change, which has been used as a model for a numerous effective interventions. It suggests that linear progression is a possible but relatively rare phenomenon; behavior change involves a movement through a series of discrete stages in a "spiral" pattern, often regressing to an earlier stage. According to the model, each time people relapse they learn from their mistakes and may try something different, more effective in future efforts [9]. TTM involves four primary constructs in explaining health behavior change: stages of change, processes of change, decisional balance and self-efficacy [10,11,8]. As far as Decisional Balance (DB) is concerned, the model examines how the individual weighs up the costs and benefits of a particular action [12,13]. Velicer and his colleagues [13] developed a measure of pros and cons of smoking adopted by Janis & Mann's research [14]. DB changes as smokers move through the five stages of change (precontemplation, contemplation, preparation, action, maintenance). During precontemplation (not intending to make any changes), the perceived benefits of smoking outweigh the perceived negatives. As the individual progresses into the action (actively engaging in a new behavior) and the maintenance (sustaining the change over time) stages, the negative perceptions of smoking overtake the positive. Smokers in the contemplation (considering a change) stage should perceive almost equal pros and cons [13]. The construct of DB refers to a cognitive activity of the TTM that has received special attention. It has been applied in studying a number of health related behaviors such as smoking [15,16,11], condom use [17] and mammography adoption [18].

Prior research has also suggested that informing smokers about health problems that smoking can cause may not be effective because smokers use cognitive-dissonance-reducing techniques [19,20]. According to the Cognitive Dissonance (CD) theory [21] the possession of inconsistent cognitions creates psychological discomfort, which motivates people to alter their cognitions (beliefs, attitudes) and behaviors to restore consistency. The emotional state of dissonance may occur when there is inconsistency between two beliefs or between a belief and a behavior. The individual will try to resolve the unpleasant dissonance by changing the belief, by changing the action or by "rationalizing" the action.

Festinger's theory has been widely used in many health related fields such as eating disorders [22], sex practices [23] and smoking [19,24,25]. Oldman [20] suggested that there are at least five models through which smokers try to reduce the conflict between smoking and concern for health: a. by rationalizing the health issue, b. by "statistical" rationalization of the health issue, c. by modifying smoking behavior, d. by denying the authority of anti-smoking information, and e. by acknowledging the risks attached to smoking.

The aim of the present study was to explore Greek habitual smokers' "readiness" (intention and self appraised capability) to quit smoking using the theoretical frameworks of DB and CD, two crucial decision making processes useful to designing interventions aiming at tobacco harm reduction. Four groups of smokers, defined according to their intention and appraisal of capability to quit smoking, were contrasted. After assessing the frequency of these states among smokers, and their possible associations with age, gender, education and smoking history, two specific hypotheses were empirically tested. First, compared to people who are ready to quit smoking, those who are not ready would perceive more benefits of smoking and this last group would record more perceived pros than cons. Second, the degree to which excuses are employed to minimize the CD will vary according to intention and perceived capability to quit smoking. We expect that the points that this study will reveal employing the approaches of the DB and CD will be instrumental in designing effective programs for tobacco harm reduction.

## Methods

Respondents were 401 people (195 women, 206 men) ranging in age from 18 to 60 years (mean = 27.5, S.D. = 9.2 years). All were habitual smokers with a self-reported average daily consumption of between "10 to 20" and "40 or more" cigarettes per day. All respondents were approached at public places (bus and train stations, airport, bars, cafeterias and public services' waiting rooms) in Thessaloniki, the second biggest city in population in Greece. Participation in the study was voluntary and no payment was made to the respondents. After a short introduction about the study, they were asked to complete an anonymous questionnaire about smoking. Non-respondent were 45 individuals (10.3%). According to respondents' answers regarding intention and self appraised capability to quit smoking they were allocated into four different groups.

All respondents were asked to complete a set of self-report measures, which included the short form of the DB Inventory [13] and an instrument based on the five models about reducing CD, proposed by Oldman [20]. Equivalent meaning for the Greek adaptations of the two instruments was ensured by back translation. Cronbach's alpha coefficients for the two scales were 0.54 and 0.63 respectively.

The short form of the DB measure was composed of a 6-item scale weighing pros (e.g. "smoking cigarettes relieves tension"), and cons (I'm embarrassed to have to smoke) of smoking. Items were rated on a five-point Likert scale ranging from "not important" to "extremely important". Scores for cons were subtracted from the sum of scores for pros to produce the aggregate DB score.

The scale based on the five models proposed by Oldman [20] was composed of 10 items which record the degree of agreement or disagreement with statements-"excuses" referring to the habit of smoking (e.g. "there is too much fuss being made about the risks related to smoking"). Items were rated on a seven-point Likert scale ranging from "strongly agree" to "strongly disagree". The lower the mean of the 10 scores the higher the degree to which excuses were employed to reduce CD. Additionally, in order to confirm the existence of CD, respondents were asked about their attitude toward smoking.

The questionnaire also included a part regarding demographic information such as age, gender, education etc. as well as information referring to smoking (heaviness, duration, quitting attempts, etc.).

## Results

The four groups differed in their "readiness" to quit smoking. Group A consisted of 94 subjects (23%), who intended and felt capable to quit and Group B consisted of 65 subjects (16%), who intended but did not feel capable to quit. Group C consisted of 90 subjects (22%), who claimed that they are capable to quit but they did not intend to and Group D consisted of the 152 subjects (38%), who stated that neither intended nor felt capable to quit. As far as the TTM is concerned, subjects in these four groups were interspersed at the precontemplation (no intention to make any changes) and the contemplation stages (considering a change). Whereas Group D subjects were clearly at the precontemplation stage, subjects from the remaining groups were dispersed between the two stages, with Group A subjects approaching the most the contemplation stage, Group C subjects approaching the less the contemplation stage and Group B falling in intermediary levels. For the statistical analysis chi-square tests as well as analysis of variance was used. In the latter case it was checked that the relevant distributions were not deviating from normality substantially and that the homoscedasticity assumption was met (homogeneity of variances tests were non significant in all cases). Age, gender and education were not found to be associated with readiness to quit smoking. About half of the respondents were women (49%) and the gender ratio was similar in the four groups (*X*^2 ^(3) = 3.38, *P *= .336). The age of our sample ranged from 18 to 60 years while most of them (72%) were in the 20 to 35 years range. On the average, age did not differ significantly between groups (*F *(3, 397) = .88, *P *= .451). Educational level was also similar in the four groups (*X*^2 ^(15) = 9.35, *P *=.859). In the entire sample, a small percentage (12%) had up to 9 years of schooling, 36% were high school graduates, 24% were university students while the rest had had higher education.

Smoking status was estimated on the basis of daily consumption of cigarettes. Most of the participants (51%) belonged to the "light smokers" group, while only 16% smoked more than 30 cigarettes per day. Duration of smoking ranged from a few months to 40 years with mean duration of 9.3 years (S.D. = 7.5). The majority of the participants (59%) had never tried to quit smoking and most of the "quit attempters" had only tried once. The four groups did not differ in "heaviness" of smoking (*X*^2 ^(9) = 6.21, *P *= .718) or duration (*F*(3, 397) = .31, *P *= .819). However, they did differ in respect of the previous quit attempts (*X*^2 ^(9) = 21.13, *P *= .012). Specifically, the significant result was mainly attributed to the difference between groups B and C. The percentage of those who had tried to quit smoking in the past was much higher among subjects in group B than among their counterparts in group C. Table 1 shows the characteristics of the respondents regarding smoking in the four groups.

**Table 1:** Smoking characteristics in groups of smokers differing in their capability and intention to quit smoking

	Groups of smokers *				
	A (n = 94)	B (n = 65)	C (n = 90)	D (n = 152)	**Entire sample**
**Duration of smoking (years)**					
Mean (SD)	8.8 (7.6)	9.3 (6.4)	9.1 (7.4)	9.7 (8.0)	9.3 (7.5)
**Smoking status**					
10–20 cigarettes per day	50 (53%)	38 (58%)	46 (51%)	70 (465)	204 (51%)
20–30 cigarettes per day	29 (31%)	20 (31%)	31 (34%)	52 (34%)	132 (33%)
30–40 cigarettes per day	12 (13%)	5 (8%)	10 (11%)	19 (13%)	46 (11%)
> 40 cigarettes per day	3 (3%)	2 (3%)	3 (3%)	11 (7%)	19 (5%)
**Quitting attempts in the past**					
none	55 (58%)	25 (39%)	61 (68%)	94 (62%)	235 (59%)
1	13 (14%)	15 (23%)	12 (13%)	29 (19%)	69 (17%)
2	10 (11%)	13 (20%)	10 (11%)	18 (12%)	51 (13%)
3 or more	16 (17%)	12 (18%)	7 (8%)	11 (7%)	46 (11%)

*Groups differing in terms of capability and intention to quit smoking

Group A: perceived capability (Y) and intention (Y)

Group B: perceived capability (N) and intention (Y)

Group C: perceived capability (Y) and intention (N)

Group D: perceived capability (N) and intention (N).

Our findings provided support to the "weighting pros and cons" hypothesis, as the four groups were found to differ significantly (*F *(3, 397) = 3.56, *P *= .014) in their DB scores. Group means presented a linear trend (weighted linear term: *F*(1, 397) = 9.50, *P *= .002, deviation term: *F*(2, 397) = .59, *P *= .556), that is the more ready the smokers were to quit the less the pros of smoking counterbalanced the cons (see Table 2). Actually, only for smokers who did not consider a change in their habit (group D), namely those clearly at the precontemplation stage, the decisional balance was positive, while smokers in the other groups perceived almost equal pros and cons.

**Table 2:** Decisional Balance and Cognitive Dissonance in the four groups of smokers

**Decisional Balance**	n	Mean	Std. Deviation	95% Confidence Interval for Mean	
Groups of smokers*				Lower Bound	Upper Bound
A	94	-.13	1.34	-.40	.15
B	65	.10	1.29	-.22	.42
C	90	.07	1.28	-.19	.34
D	152	.40	1.25	.20	.60
Total	401	16	1.30	.029	.28

Cognitive Dissonance**	n	Mean	Std. Deviation	95% Confidence Interval for Mean	

Groups of smokers*				Lower Bound	Upper Bound
A	67	4.11	1.04	3.86	4.37
B	56	3.61	1.06	3.32	3.89
C	70	4.04	1.02	3.79	4.28
D	122	3.86	.97	3.69	4.04
Total	315	3.91	1.02	3.80	4.02

*Groups differing in terms of capability and intention to quit smoking

Group A: perceived capability (Y) and intention (Y)

Group B: perceived capability (N) and intention (Y)

Group C: perceived capability (Y) and intention (N)

Group D: perceived capability (N) and intention (N).

** refers to the 315 smokers with negative attitude towards smoking. Scores reflect the degree to which excuses were not employed to reduce CD

Regarding CD, 315 subjects (79%) were considered cognitively dissonant, since they had a negative attitude towards smoking (stated that they consider smoking a bad habit) although they did smoke. The percentage of cognitively dissonant individuals did not differ significantly between the four groups (*X*^2 ^(3) = 5.48, *P *= .140). Comparisons of the four groups revealed differences on the level of efforts to moderate the dissonance between attitude and action (*F *(3, 311) = 3.03, *P *= .029). Post hoc testing showed that the significant result was attributed to the difference between groups B and A. Specifically, those who intended but did not feel capable to quit used more "excuses" compared to those that intended and felt capable. The remaining two groups of smokers who did not intend to quit, did not differ significantly neither from A nor from B.

As the ratio of people with previous attempts of quitting smoking was not the same in the four groups, differences between groups in DB, as well as in CD, were examined, after taking into account this factor. Two-way analysis of variance for DB revealed that the effect of previous attempts to quit smoking was not significant, while for CD a significant effect was found (*F*(1, 306) = 7.44, *P *= .007). Smokers who had past unsuccessful quit attempts, recruited fewer "excuses" to justify the continuation of the smoking habit (mean = 4.08, SD = 1.03 versus mean = 3.79, SD = 1.00, for those who had never tried to quit smoking). The group effect in CD was still significant, after adjustment (*F*(3, 306) = 3.85, *P *= .010). Post hoc testing of estimated group means of CD, after adjustment for previous attempts confirmed that the significant result was attributed to the difference between smokers who intended but did not feel capable to quit, and those that intended and felt capable of quitting.

## Discussion

As predicted by the Decisional Balance theory and in consistency with past research [10,11] our findings support the hypothesis that people who are not ready to quit smoking perceive more benefits of smoking than negatives and come to the conclusion that pros are more than cons, so they can continue smoking. Clearly, the processes described by Velicer and his colleagues [13] of thinking about the positives and negatives of smoking and coming to some conclusion are confirmed as important mediators in the decision making process for smoking cessation.

Regarding CD, those who had previous unsuccessful attempts to quit smoking were found to employ fewer excuses, compared to those who had never tried to. It seems that the quit attempters felt less uncomfortably with their dissonance compared to those who had never tried to quit, since they had tried to resolve it by changing their action ("I have done what I ought to do"). Moreover, the quit attempters were more likely to intend but not feel capable to quit and less likely not to intend but feel capable to. It appears that unsuccessful quit attempts discourage smokers and make them feel that they cannot be successful in future attempts, whereas those who had never tried to quit feel they can quit whenever they decide to. This finding is in line with TTM theory, pertaining that people in a change process move through stages, often regress to an earlier stage and whenever they relapse, learn from their mistakes and move on toward success, since past attempters employ fewer "excuses". What they have learned is not to lie to themselves, and have reached a level of awareness of their "problem". However, this theory cannot account for the fact that past attempters are more likely to feel incapable to succeed. This last finding is in line with the "Learned Helplessness" model (LHM) [26-28], as far as the prediction of smoking behavior is concerned. Regarding human behavior, LHM holds that people who face continued noncontingent failure in achievement situations reveal a belief of powerlessness to control the outcomes and show a worsening performance [29,30].

Some limitations of the current work should be acknowledged. The cross-sectional design of the study may have limited the statistical power of our findings. As far as DB is concerned, a possible limitation could be the use of the short form of the instrument. The pros and cons of smoking may form a complex construct that is less reliably measured by a three-item scale each.

The findings concerning Decisional Balance should direct future interventions in increasing the importance of the pros of quitting and/or decreasing the importance of the cons. Further investigation, on the "weighting pros and cons" process, might be proved fruitful, especially if future efforts include exploration of both the role of stages of change and readiness to quit. Since the mechanism of employing excuses to relieve CD was not found to be much in use among smokers, future research should identify the different mechanisms that smokers resort to in order to relieve conflict so that more effective campaigns can be designed.

Additionally, effective interventions should not be limited to urging people to quit without at the same time providing strong support to succeed in quitting and sustain the smoke-free condition, as unsuccessful quit attempts seem to render the attempter powerless and persuade him that the goal is unreachable.

## Conclusion

Support for the DB theory is provided, as the smokers weigh the pros and cons of smoking before they reach a decision on quitting or continuing. On the other hand, smokers do not resort to extensive employing of "excuses" in order to reduce the conflict between smoking and concern for health, with those who had previous unsuccessful attempts to quit smoking, employing fewer excuses compared to those who had never tried to. Moreover, the unsuccessful quit attempters are more likely to intend but not feel capable to quit. It appears that unsuccessful quit attempts discourage smokers and make them feel that they cannot be successful in future quit attempts. Effective interventions aiming harm reduction should take into account the detrimental effect of past unsuccessful quit attempts. If we are to bring forth tobacco harm reduction by proposing quitting smoking we must understand that launching campaigns promoting overall quitting might prove detrimental to the implementation of our goal. It proves crucial to sustain such efforts by programs designed to ensure success once for all.

## Competing interests

The author(s) declare that they have no competing interests.

## Authors' contributions

M.L., PhD conceived of the study and its design, the collection of data, drafting the article and making the final edits, and giving final approval for publication.

V.B., Ms mentored Ms M.L. in the study conceptualization, design, data collection, manuscript preparation, giving final approval for publication.

J-O.S., PhD participated in the design and methodology of the study, organization and statistical analysis of the data, editing the manuscript, and giving final approval for publication.
